# The Extracellular Matrix in Glioblastomas: A Glance at Its Structural Modifications in Shaping the Tumoral Microenvironment—A Systematic Review

**DOI:** 10.3390/cancers15061879

**Published:** 2023-03-21

**Authors:** Salvatore Marino, Grazia Menna, Rina Di Bonaventura, Lucia Lisi, Pierpaolo Mattogno, Federica Figà, Lal Bilgin, Quintino Giorgio D’Alessandris, Alessandro Olivi, Giuseppe Maria Della Pepa

**Affiliations:** 1 Department of Neuroscience, Neurosurgery Section, Università Cattolica del Sacro Cuore, 00168 Rome, Italyalessandro.olivi@policlinicogemelli.it (A.O.); 2 Department of Neurosurgery, Fondazione Policlinico Universitario Agostino Gemelli IRCCS, 00168 Rome, Italy; 3 Dipartimento di Sicurezza e Bioetica, Università Cattolica del Sacro Cuore, IRCSS-Fondazione Policlinico Universitario Agostino Gemelli, 00168 Rome, Italy

**Keywords:** glioblastoma, extracellular matrix, cancer, tumor microenvironment

## Abstract

**Simple Summary:**

The new trends in the research on glioblastomas (GBMs) are focusing on understanding the crosstalk between proper neoplastic tissue and its microenvironment. Against this background, the extracellular matrix (ECM) has been classically linked to a purely structural role. However, it has recently become clear that it actively shapes the functional responses of cells to their environment as well. While many components of the ECM have been isolated and characterized, its modifications in the specific setting of GBMs have only been recently explored in the literature. The aim of this paper is to provide a systematic review on the topic and to assess the ECM’s role in shaping tumoral development.

**Abstract:**

Background and aim: While many components of the ECM have been isolated and characterized, its modifications in the specific setting of GBMs have only been recently explored in the literature. The aim of this paper is to provide a systematic review on the topic and to assess the ECM’s role in shaping tumoral development. Methods: An online literature search was launched on PubMed/Medline and Scopus using the research string “((Extracellular matrix OR ECM OR matrix receptor OR matrix proteome) AND (glioblastoma OR GBM) AND (tumor invasion OR tumor infiltration))”, and a systematic review was conducted in accordance with the PRISMA-P guidelines. Results: The search of the literature yielded a total of 693 results. The duplicate records were then removed (n = 13), and the records were excluded via a title and abstract screening; 137 studies were found to be relevant to our research question and were assessed for eligibility. Upon a full-text review, 59 articles were finally included and were summarized as follows based on their focus: (1) proteoglycans; (2) fibrillary proteins, which were further subdivided into the three subcategories of collagen, fibronectin, and laminins; (3) glycoproteins; (4) degradative enzymes; (5) physical forces; (6) and glioma cell and microglia migratory and infiltrative patterns. Conclusions: Our systematic review demonstrates that the ECM should not be regarded anymore as a passive scaffold statically contributing to mechanical support in normal and pathological brain tissue but as an active player in tumor-related activity.

## 1. Introduction

The new trends in the research on glioblastomas (GBMs) are focusing more and more on the understanding of the crosstalk between proper neoplastic tissue and its microenvironment (TME). The microenvironmental contribution seems to be critical in tumor development, progression, relapse, and resistance to therapies. A definition of the “seed and soil” approach has recently been introduced to describe the glioblastoma landscape: the TME, mainly constituted by inflammatory cells (microglia, monocytes, and macrophages) and stem cells, acts as a fertile “soil” interacting with the “seed”, which is represented by proper neoplastic glial cells [1]. Against this background, the extracellular matrix (ECM) has been classically linked to a mere structural role. However, it has recently become clear that it actively shapes the functional responses of cells to their environment as well. Therefore, the ECM should not be regarded anymore as a passive scaffold statically contributing to mechanical support but as an active player in tumor-related activity: it shows dynamic “structural” modifications and interactions under the pressure of the dysregulated TME, it performs active crosstalk with the inflammatory and stem compartment, and it is able to influence cellular migration [2,3,4,5]. While many components of the ECM have been isolated and characterized, its modifications in the specific setting of GBMs have only been recently explored in the literature. The aim of this paper is to provide an updated systematic review on the topic and to assess the ECM’s role in shaping tumoral development.

## 2. Materials and Methods

The study presented herein was conducted in accordance with the PRISMA-P (Preferred Reporting Items for Systematic Review and Meta-Analysis Protocols) guidelines [2]. An online literature search was launched on PubMed/Medline and Scopus using the research string “((Extracellular matrix OR ECM OR matrix receptor OR matrix proteome) AND (glioblastoma OR GBM) AND (tumor invasion OR tumor infiltration))”; research was last conducted in September 2022. Two authors, S.M. and F.F., independently conducted the abstract screening for eligibility. Any discordance was solved through consensus with a third senior author, G.M.D.P. No restrictions on date of publication were made. Exclusion criteria were as follows: studies published in languages other than English and meta-analyses. A systematic abstract screening of the references (forward search) was performed to identify additional records.

## 3. Results

**Figure 1:**
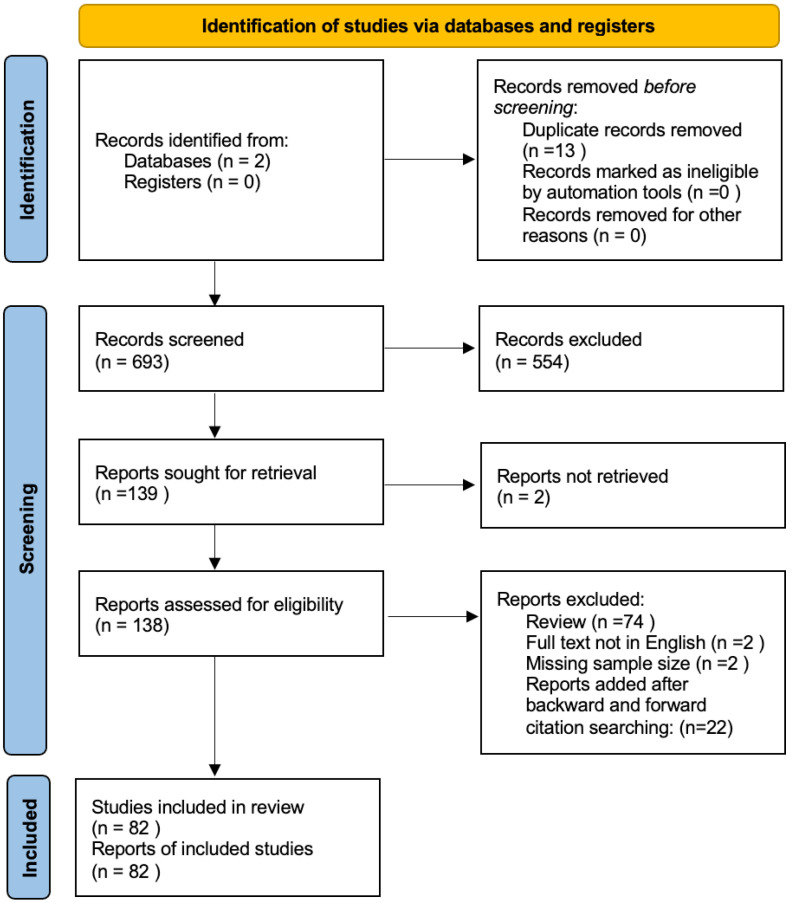
Prisma flowchart summarizing the results of our research.

The search of the literature yielded a total of 693 results. The duplicate records were then removed (n = 13), and the records were excluded via a title and abstract screening; 138 studies were found to be relevant to our research question and were assessed for eligibility (Figure 1). Upon a full-text review and forward search, 82 articles were finally included.

The reviewed papers were further divided into six categories based on their focus:

(1) Proteoglycans (Table 1);
(2) Fibrillary proteins, which were further subdivided into three subcategories: collagen (Table 2a), fibronectin (Table 2b), and laminins (Table 2c);
(3) Periostin (Table 3);
(4) Glycoproteins (Table 4);
(5) Degradative Enzymes (Table 5);
(6) Physical forces (Table 6);
(7) Glioma cell and microglia migratory and infiltrative patterns (Table 7).

**Table 1:** Proteoglycans.

Author	Year	Type of Study	Sample Size	Main Findings
Logun, M.T. [6]	2017	In vitro cell culture	1 human cell line	CS-GAGs directly induce the enhanced cell migration and haptotaxis of glioma cells.
Logun, M.T. [7]	2019	In vitro cell culture	1 murine cell line	GAG promotes GBM cell invasion.
Schrappe, M. [8]	1991	Human GBMs and monoclonal antibodies	5 human GBMs	Human GBMs specifically express a chondroitin sulfate proteoglycan that is recognized by monoclonal antibodies and is localized on the glioma cell surface.
Kim, Y. [9]	2018	Mathematical model		High-molecular-weight CSPGs can regulate the exodus of local reactive astrocytes from the main tumor lesion, leading to an encapsulation of noninvasive tumors and an inhibition of tumor invasion.
Silver, D.J. [10]	2013	In vitro cell culture and xenograft	3 human cell lines	Microenvironmental glycosylated chondroitin sulfate proteoglycans inversely correlate with the invasive character of human gliomas.
Onken, J. [11]	2013	In vitro cell culture and siRNA	2 human cell lines	Versican isoform V1 is a proliferation-enhancing and promigratory molecule in high-grade glioma in vitro.
Tran, V.M. [12]	2018	In vitro cell culture	4 human cell lines and 2 murine cell lines	HPSE has a role in tumor invasion, acting on HSPGs on the cell surface.
Su, G. [13]	2006	In vitro cell culture	5 human cell lines	HSPGs have a great capability to promote FGF-2 signaling. The enhanced HSPG activity correlated with a high level of expression of Gpc-1 and structural HSGAG alterations.
Watanabe, A. [14]	2005	Reverse transcription PCR	10 glioma cell lines, 2 GBM specimens, and 2 normal brain specimens	Syndecan-1 is a key molecule in the motility of cells; it is crucial in coupling the organization of fascin spikes in response to a physiological extracellular ligand, TSP-1, and an overexpression of syndecan-1 in a heterologous cell type is sufficient for causing a dramatic enhancement of cell spreading and formation of fascin spikes in response to TSP-1. It is significantly expressed by GBM cells.
Chen, J.E. [15]	2018	matrix-bound HA and xenograft GBM population	1 patient-derived xenograft GBM population	GBM migration is strongly influenced by HA molecular weight.
Hayen, W. [16]	1999	hyaluronan-containing fibrin gels and GBM cell lines	1 GBM cell line	In complex three-dimensional substrates, the predominant effect of hyaluronan on cell migration might be indirect and requires modulation of fibrin polymerization.
Chen, J.W.E. [17]	2018	In vitro cell culture and hyaluronan synthesis inhibition	2 cell lines	GBM cells under hypoxia show invasive behavior. The lack of matrix-bound HA affects GBM response by inducing compensatory HA secretion of this essential cell adhesive biomolecule, which is associated with increased GBM invasion.
Chen, J.W.E. [18]	2022	culture of patient-derived GBM cells		CD133+ GBM subpopulation increases in response to both hypoxia and matrix-bound hyaluronan.
Akiyama, Y. [19]	2001	Cell culture and surgical specimen	Antibody blockage	HA-receptors contribute to brain tumor adhesion, proliferation, migration, and biological features.
Pibuel, M.A. [20]	2021	In vitro cell culture and hyaluronan synthesis inhibition	2 human cell lines	4MU markedly inhibits cell migration and induces senescence in human GBM cell lines; 4MU modulates the expression and the distribution of CD44, RHAMM, and MMP-2.
Tsatas, D. [21]	2002	In vitro cell culture and antibody blockage	4 glioma cell lines	Hyaluronan induces genes encoding matrix-degrading enzymes (plasminogen cascade).
Zhang, H. [22]	2022	In vitro cell culture and hyaluronan synthesis inhibition	3 cell lines	HA was found to mediate glioma proliferation, progression, and invasion; it potentially promoted macrophage recruitment and M2 polarization through the IL-1/CHI3L1 and TGF-b/CHI3L1 axes.

**Table 2:** (a) Collagen, (b) fibronectin, and (c) laminins.

Author	Year	Type of Study	Sample Size	Main Findings
(a)				
Calori, I.R. [23]	2022	GBM cell lines and type I collagen	4 cell lines	The enzymatic cleavage of collagen affects spheroid morphology and increases cell migration while maintaining cell viability.
Wang, Y. [24]	2022	GBM cell lines and COL1A2 siRNA	3 cell lines	COL1A2 plays an important role in driving GBM progression. COL1A2 inhibition attenuates GBM proliferation by promoting cell cycle arrest.
Chintala, et al. [25]	1996	In vitro cell culture	4 cell lines	Collagen type II is involved in migration and invasion of glioblastoma cells.
Mammoto, T. et al. [26]	2013	In vitro cell culture and antibody blockage	3 cell lines	D-penicillamine decreases collagen expression, disrupts collagen structure in tumors, and inhibits brain tumor growth.
Senner, V. [27]	2008	In vitro cell culture and siRNA	5 cell lines	Glioma cell lines can utilize collagen type XVI as a substrate for adhesion.
Huijbers, I.J., et al. [28]	2010	Human cell culture and antibody blockage	79 gliomas	Fibrillar collagens are extensively deposited in GBMs; the collagen type I internalization receptor Endo180 is both highly expressed in these tumors and serves to mediate the invasion of tumor cells through collagen-containing matrices.
Lin, J. [29]	2021	In vitro cell culture and xenograft	58 gliomas	P4HA2 is a prognostic marker and exerts oncogenic functions to promote the malignancy of gliomas (grade II to grade IV). The underlying mechanism may be regulating the collagen-dependent PI3K/AKT signaling pathway.
Jiang, X. [30]	2017	In vitro cell culture and xenograft	2 cell lines	HSP47 promotes GBM stem-like cell survival by modulating tumor microenvironment ECM through TGF-β pathway.
(b)				
**Author**	**Year**	**Type of Study**	**Sample Size**	**Main Findings**
Ohnishi, T. [31]	1998	In vitro human cell culture and antibody blockage	9 GBM samples	Fibronectin concentration seems to be higher in tumor cells and promotes migration of glioma cells.
Chintala, S.K. [32]	1996	In vitro human cell culture and antibody blockage	13 GBM samples	Glioblastoma cells produce collagen type IV, laminins, and fibronectin.
Caffo, M. [33]	2004	In vitro human cell culture and antibody blockage	6 GBM samples	Integrins appear to be of great interest in GBM treatment either as targeted therapies, drug-delivering vectors, or diagnostic tools for tumor imaging.
Serres, E. [34]	2013	In vitro human cell culture and antibody blockage	3 GBM lines	FN produced by tumor cells has a role in GBM pathophysiology.
Sengupta, S. [35]	2010	Xenograft and siRNA	Murine glioma cell line	Fibronectin silencing aborts integrin signaling in GL261 cells and fails to initiate Src kinase and STAT3 activity, thus aggressively reducing survivin expression.
Huang, J.M. [36]	2006	In vitro cell culture and GBM cell line	1 GBM cell line	The interaction between beta1-integrin and FN may stimulate U251MG cell migration, changing the structure of the microfilament skeleton and the number of pseudopodia. Beta1-integrin may play a role in the LN-mediated in vitro invasion of U251MG cells.
Yu, S. [37]	2020	Xenograft and siRNA	3 cell lines	GBP2 dramatically promotes GBM tumor growth and invasion in mice and significantly reduces the survival time of the mice with a tumor.
Kabir, F. [38]	2022	Mathematical model—bioinformatics model	7 data sets	FN1 has prognostic value in GBMs.
(c)				
**Author**	**Year**	**Model Used**	**Sample Size**	**Main Findings**
Tysnes, B.B. [39]	1999	Xenograft	5 GBM samples	Laminins can be produced by GFAP positive cells during glioma cell invasion in humans.
Caffo, M. [33]	2004	In vitro human cells and antibody blockage	6 GBM samples	Integrins appear to be of great interest in GB treatment either as targeted therapies, drug-delivering vectors, or diagnostic tools for tumor imaging.
Sun, T. [40]	2022	Xenograft culture	107 GBMS samples	Inhibition of the vascular BM component laminin-411, which is produced by tumor cells like many other tumor ECM components, disrupts the perivascular CSC niche, negatively affects CSCs, and may enhance the efficacy of glioma therapy.
Khazenzon, N.M. [41]	2003	In vitro human cell culture and antibody blockage	2 cell lines	Laminin-8 may play an important role in glioma invasion.
Gamble, J.T. [42]	2018	Xenograft culture	1 cell line	Laminin alpha 5 significantly lowers the invasion of mobile U251MG cells.

**Table 3:** Periostin.

Author	Year	Model Used	Sample Size	Main Findings
Wang, H. [43]	2013	Frozen glioma tissue and microarray	220 frozen glioma tissues	The expression levels of POSTN are relative to glioma grade progression and are inversely correlated with overall survival in high-grade glioma patients.
Landré, V. [44]	2016	In vitro human cell culture, plasmids, and antibodies	2 cell lines	TAp73 controls glioblastoma cell invasion by regulating the expression of the matricellular protein POSTN.
Ouanouki, A. [45]	2018	In vitro human cell culture	1 cell line	Periostin acts as a central element in TGF-β-induced EMT.

**Table 4:** Glycoprotein—tenascin.

Author	Year	Model Used	Sample Size	Main Findings
Xia, S. [46]	2016	Xenograft culture	2 human cell lines	TNC expression levels or gene copy numbers do not significantly affect patient survival, and TNC knockdown cells are more sensitive to antiproliferative strategies.
Hirata, E. [47]	2009	Xenograft culture	1 human cell line	Endogenous tenascin facilitates GBM cell invasion by regulating focal adhesion, and, therefore, GBMs with higher Tenascin C expression have a more aggressive behavior.
Zhang, J.F. [48]	2019	In vitro human cell culture and siRNA	2 cell lines	IL-33/NF-κB/TNC supports cancer progression.
Sarkar, S. [49]	2015	Xenograft culture	7 human cell lines	TNC is a promoter of the invasiveness of BTICs through a mechanism involving ADAM-9 proteolysis via the c-Jun NH2-terminal kinase pathway.
Sarkar, S. [50]	2006	In vitro human cell culture	2 cell lines	Tenascin-C is a favorable substrate for glioma invasiveness; its effect is mediated through MMP-12.
Mai, J. [51]	2002	In vitro human cell culture		Cathepsin B and Tenascin-C are highly expressed in malignant anaplastic astrocytomas and glioblastomas when compared to normal brain tissues and are associated with tumor neovessels.

**Table 5:** Degradative enzymes.

Author	Year	Model Used	Sample Size	Main Findings
Li, Q. [52]	2016 Mar	Data set and genome mRNA	23 types of MMPs and 305 gliomas	Patients expressing MMP9 may have a longer survival and may benefit from temozolomide chemotherapy.
Lakka, S.S. [53]	2004	In vitro human cell culture and siRNA	1 cell line	Simultaneous RNAi-mediated targeting of MMP-9 and cathepsin B has potential application in the treatment of human gliomas.
Kargiotis, O. [54]	2008	In vitro human cell culture and xenograft	4 cell lines	MMP-2 inhibition induces apoptotic cell death and suppresses tumor growth.
Schuler, P.J. [55]	2012	Xenograft	1 cell line	uPA, uPAR, MMP-2, and MMP-9 play an important role in GBM growth.
Sun, J. [56]	2019	In vitro human cell culture	12 GBMs	TRAF6 and MMP9 have higher expression in GBMs compared to adjacent tissues. High expression of TRAF6 and MMP9 is significantly associated with unfavorable prognoses.
Zhao, Y. [57]	2008	In vitro human cell culture and recombinant protein	1 cell line	uPA directly cleaves the latent form of MMP-9 both at the N- and C-terminus, and this novel activation pathway promotes U1242 GBM cell invasion.
Chang, L. [58]	2015	In vitro human cell culture	1 cell line	The hedgehog signaling pathway promotes the invasion and migration of GBM cells by enhancing MMP-2 and MMP-9 expression via the PI3K/AKT pathway.
Zheng, Q. [59]	2019	In vitro human cell culture	2 cell lines	IL-17A promotes GBM cell migration and invasion via PI3K/AKT signaling pathway.
Das, G. [60]	2011	Human cell culture and antibodies	2 cell lines	Rictor bridges two major pathways—Akt (PKB)/mTOR and Raf-1-MEK-ERK—for regulation of MMP-9 activity and invasion of glioma tumor cells.
Djediai, S. [61]	2021	Human cell culture and RNA isolation	1 cell line	MT1-MMP and TGF-β mediate EMT-like induction in glioblastoma cells.
Zhai, Y. [62]	2022	Immunostaining with rabbit monoclonal antibodies	214 gliomas	MT1-MMP, β1-integrin, and YAP1 are prognostic biomarkers.
Held-Feindt, J. [63]	2005	Human GBM samples, GBM cell lines, and RT-PCR	4 GBM cell lines	In human glioblastomas, secretory proteases, such as ADAMTS4 and ADAMTS5, are expressed at the mRNA and protein levels in considerable amounts.
Siney, E.J. [64]	2017	Excised high-grade glioma and antibody inhibition	12 excised GBMs	ADAM10 and ADAM17 inhibition selectively increases GSC migration, and the migrated GSCs exhibit a differentiated phenotype.

**Table 6:** Physical forces.

Author	Year	Model Used	Sample Size	Main Findings
Herrera-Perez, M. [65]	2015	In vitro human cell culture in 3D matrix		Migration of glioblastoma stem cells is reduced by the presence of hyaluronan.
Ulrich, T.A. [66]	2009	In vitro human cell culture	2 cell lines	Increasing ECM rigidity can induce a cascade of phenotypic changes in human glioma cells, which includes increased cell spreading, faster motility, and enhanced proliferation.
Kaufman, L.J. [67]	2005	Human cells encapsulated in 3D hydrogel	1 cell line	GBM tumors are affected significantly by the total collagen concentration in the gel, and there are distinct growth patterns in low- and high-concentration collagen type I gels. Specifically, increasing concentrations of collagen type I correlate positively with invasion but negatively with MTS growth.
Wang, C. [68]	2014	In vitro human cell culture	15 primer sequences	Matrix stiffness modulates GBM progression.
Lim, E.J. [69]	2018	In vitro cell culture and siRNA	1 cell line	tMSLCs, as stromal cells, provide force-mediated proinvasive ECM remodeling in the GBM microenvironment.
Pu, W. [70]	2020	In vitro human cell culture	2 cell lines	MPs play pivotal roles in the invasiveness of GBMs by degrading the surrounding tissue, activating signal transduction, and releasing ECM-bound growth factors.

**Table 7:** Glioma cell and microglia migratory and infiltrative patterns.

Author	Year	Model Used	Sample Size	Main Findings
Koh, I. [71]	2018	In vitro human cell culture	1 cell line	MMP9 and HAS2 are highly upregulated in pdGCs cultured within the pdECM. In fact, both MMPs and HASs have been implicated in playing crucial roles in GBM invasiveness.
Herrera-Perez, M. [65]	2015	In vitro human cell culture		GSC migration is not limited to a unique migration mode that is usually observed in in vitro studies but is able to concomitantly exhibit multiple migration modes (collective and single) as a response to the heterogeneity of the environment.
Rao, S.S. [72]	2013	In vitro human cell culture and 3D matrix	1 cell line	GBM migration is an inverse function of HA concentration, with HA impeding and eventually stopping cell movement.
Cui, Y. [73]	2020	In vitro human cell culture and 3D matrix	1 cell line	HA addition to the collagen culture environment induces many changes consistent with the amoeboid migratory phenotype, including rounded morphology, squeezing or gliding motility, cortical actin expression, reduced cell–fiber interactions, and reduced integrin expression.
Hirata, E. [74]	2012	Xenograft and shRNA	3 cell lines	Zizimin1 appears to play an important role in the formation of multiple pseudopodia and invasion of the brain parenchyma.
Lively, S. [75]	2013	Cultured rat microglial cells	1 cell line	Microglial cells migrate during CNS development and after CNS damage or disease.
de Vrij, J. [76]	2015	Human cell culture	2 cell lines	EVs are mechanisms for GBMs to use to induce MT1-MMP expression in GBM associated microglia, supporting tumor growth.
Gabrusiewicz, K. [77]	2011	Murine glioma cells and xenograft	1 cell line	Resident microglia and blood-derived macrophages contribute to a pool of glioma-infiltrating immune cells and regulate tumor angiogenesis and invasion, which are essential for glioma progression.
Bettinger, I. [78]	2002	Murine glioma cell and mouse microglial cell cultures	1 cell line	Microglial cells promote the invasive phenotype of diffuse astrocytoma cells.
Markovic, D.S. [79]	2005	Murine glioma cell and microglial cell cultures		The presence of microglia in a GBM has a protumorigenic effect.
Markovic, D.S. [80]	2009	Murine glioma cells and shRNA	1 cell line	Protumorigenic role of microglial cells is substantial and may put microglial cells into focus as a target for new brain tumor therapies. Therapeutic TLR blockade, which may be achieved with TLR subtype-specific antagonists, could serve as a future tool to attenuate microglia-promoted tumor invasion.
Wu, C.Y.J. [81]	2020	Human cell culture and poli- and monoclonal antibodies	3 cell lines	Chemokine axis in the glioma microenvironment is subject to CCL5-mediated invasion, and such regulation is facilitated by GAM activation. Restriction of calcium-dependent pathways may be pivotal in eliminating CCL5/GAM-regulated glioma invasion.
Kulla, A. [82]	2000	Human cell culture	90 gliomas	Higher numbers of tumor-infiltrating macrophagic/microglial cells are present in TN-positive areas of human gliomas; TN serves as a permissive substrate for macrophage migration and may have a certain role in modulating and possibly promoting the trafficking of cells of monocyte lineage in malignant human gliomas.
Xia, S. [46]	2016	Xenograft culture and shRNA	2 cell lines	TNC knockdown cells are more sensitive to antiproliferative strategies, which could ultimately lead to novel combinatory antitumor strategies that can target both tumor invasion and proliferation.
Hu, F. [83]	2015	Murine and human cells and animal xenograft	5 cell lines	Versican, released from gliomas, promotes tumor expansion through glioma-associated microglial/macrophage TLR2 signaling and subsequent expression of MT1-MMP.
Juliano, J. [84]	2018	Animal model and retrovirus injection		Increased density of glioma cells is correlated with increased activation of microglia.

### 3.1. Proteoglycans

Proteoglycans are a heterogeneous group of complex extracellular and cell surface macromolecules composed of a central core protein with covalently linked glycosaminoglycan (GAG) chains. Through interactions with chemokines, neurotrophins, growth factors, and the other components of the ECM, proteoglycans (PGs) play a critical role in many basic processes of the CNS, including cellular proliferation, migration, specification, synaptogenesis, plasticity, and regeneration [6]. For these reasons, it has been proposed that PGs could be involved in several aspects of tumor biology, including cell proliferation, tumor cell adhesion and migration, inflammation, and angiogenesis. Indeed, recent studies have proven that heparan sulfate proteoglycans (HSPGs) and chondroitin sulfate proteoglycans (CSPGs) are largely upregulated in GBM samples relative to normal brain tissue [7].

Chondroitin sulfate proteoglycans (CSPGs) consist of a protein core and covalently attached chondroitin sulfate side chains. It has been noticed that CSPGs and related enzymes are upregulated in the GBM microenvironment relative to normal tissue [8]. Similarly, in vitro studies have shown an upregulation of focal adhesion proteins, such as FAK and Vinculin, and a faster migration of glioma cells in oversulfated hydrogel matrices when compared to nonsulfated hydrogels. Moreover, these data suggest that CSPGs can modulate glioma invasiveness in a GAG-sulfation-dependent manner. Indeed, CSPGs show a different affinity to chemokines and chemokine receptors according to the sulfatation rate of CS-GAG [7]. Beyond the sulfatation rate, even the pure concentration of CSPGs in the microenvironment has been identified as a major parameter in the regulation of glioma cell invasiveness. In fact, low-CSPG levels in the microenvironment are associated with the downregulation of the LAR (leukocyte common antigen-related)-CSGAG complex, while high-CSPG levels induce its upregulation. LAR is a CS-GAG receptor that regulates cell adhesion to the ECM components. When LAR-CSGAG is downregulated, adhesion between the tumor cells and the ECM components is weak, therefore allowing the tumor cells to spread. On the other hand, high levels of CSPGs and, consequently, an upregulation of LAR-CSGAG induces strong adhesion, preventing the dispersion of the glioma cells. Moreover, LAR-CSGAG seems to influence the activation and migration of the microglia toward the tumor periphery [9,10].

Versican is one of the most represented proteins among the CSPGs in the ECM. An in vitro cell culture study revealed an important role of Versican in the regulation of glioma cell migration and adhesion. Indeed, the downregulation of Versican in the isoform V1 by siRNAs is associated with a significant reduction in proliferation and migration in glioblastoma cell lines, and TGF-beta 2, a well-known modulator of glioma cell invasion, was identified as the primary inductor of Versican 1 [11].

HSPGs consist of a core protein and covalently attached heparan sulfate (HS) glycosaminoglycan chains. Extensive co- and posttranslational enzymatic modifications, particularly involving the 6-O-sulfate (6OS) of glucosamine, generate great structural heterogeneity. An analysis conducted on human GBM cell lines and murine GBM cell lines demonstrated great heterogeneity in the content and in the structure of HS glycosaminoglycans between different glioma cell lines, suggesting a role in tumorigenesis and subtype differentiation. Heparanase is an enzyme involved in the biological regulation of HSPGs since it cleaves HS chains to reduce the HS chain length and to release smaller biologically active oligosaccharides. Heparanase induces the modification of the HS content and structure in the microenvironment and, therefore, is thought to be involved in GBM genesis. Indeed, a study on cell invasion into a three-dimensional matrix showed that clones with heterozygous deletions in HPSE and reduced HPSE expression exhibit a marked decrease in tumor cell invasion and cell adhesion to laminins [12].

Glypicans and Syndecans represent the most expressed families of heparan sulfate proteoglycans in the brain. Glypicans are overexpressed in the glioma microenvironment when compared to normal brain tissue and, according to the literature, can stimulate glioma growth by inducing the upregulation of the FGF-2 signal [13,14]. Similarly, the Syndecan family is overexpressed in the glioma microenvironment. Particularly, Syndecan-1 is overexpressed in almost all glioma cell lines studied and is poorly expressed in normal specimens. It has been suggested that the overexpression of Syndecan-1 in the glioma microenvironment induces tumor invasion through the upregulation of thrombospondin-1 [14].

Hyaluronan is a linear and nonsulfated GAG which can bind ECM proteins and proteoglycans, building a three-dimensional network. HA serves as a ligand for the membrane receptor CD-44 and the RHAMM and MEK/ERK signaling pathways, participating in cellular growth, cellular proliferation, and cellular differentiation. In vitro studies have demonstrated that glioma cell behavior differs on the base of the HA structure in the tumor microenvironment since hydrogel matrices containing high-molecular-weight HA (500K) showed significantly reduced invasion when compared to all other hydrogel groups (-HA, 10, and 60K) [15]. Moreover, gels resulting from fibrin polymerization in the presence of HA stimulate glioma cell migration, suggesting that HA could regulate glioma cell invasiveness by modulating the fibrin fiber architecture [16]. The recent data show that hypoxia could enhance endogenous HA production by glioblastoma cells [17] and that HA could stimulate glioblastoma growth by upregulating CD133+ GBM cell fractions [18]. Concerning HA downstream signaling inducing glioma cell migration, receptor CD-44 and RHAMM appear to be involved [19]. CD44-HA mediated cell invasion can be modulated by EGFR, a well-known receptor overexpressed in gliomas. In fact, CD-44 binds to EGFR, leading to an upregulation of urokinase-type plasminogen activator (uPA), urokinase-type plasminogen activator receptor (uPAR), and plasminogen activator inhibitor-1 (PAI-1) in response to HA [20,21]. Moreover, recent studies have suggested that HA potentially promotes macrophage recruitment and M2 polarization through the IL-1/CHI3L1 and TGF-b/CHI3L1 axes and that it also regulates the expression of PD-L1 [22]. 

### 3.2. Fibrillar Proteins

#### 3.2.1. Collagen

Normal brain tissue ECMs are poor in collagen, whereas its content in the glioma microenvironment and especially around vessels presents a large increase. Actually, different subtypes of collagen have been investigated over the years and are related to GBM invasiveness. Recent findings have shown that GBM cell lines form tight spheroids in the presence of type I collagen and that the enzymatic cleavage of collagen affects spheroid morphology and increases cell migration [23]. In particular, the collagen alpha-2(I) chain (COL1A2) was found to be upregulated in GBMs compared with normal brain tissue, and it is related to poor progression-free survival and overall survival [24]. Similarly, collagen type III was found to play a role in the modulation of migration and the invasion of glioblastoma cell lines in a dose-related manner; moreover, migration and invasion were inhibited in the presence of monoclonal type III collagen antibodies [25]. In vitro studies revealed that, under the specific conditions of physical compaction, human glioblastoma cell lines induce the expression of collagen type IV and type VI. Moreover, collagen disruptors such as β-aminopropionitrile induced the inhibition of glioblastoma growth in the mouse orthotopic brain tumor model [26]. Collagen type XVI mRNA was also found to be upregulated in glioblastoma cell lines. It seems to play a role in glioma cell adhesion to the ECM since a SiRNA knockdown resulted in decreased cell adhesion, although migration remained unchanged [27]. 

Different families of receptors and different pathways have been supposed to be involved in collagen-related GBM invasion. Functionally, Endo180 (CD280), a collagen-binding receptor overexpressed in GBMs, serves as the major collagen internalization receptor in GBMs and is critical in glioma cell invasion into the ECM [28]. Prolyl-4-hydroxylase subunit 2 (P4HA2) is a member of the collagen modification enzymes involved in the remodeling of the extracellular matrix (ECM). The main transcriptional P4HA2 level was found to be higher in glioma samples compared to normal brain tissue, and this correlates with glioma grading and patient survival; moreover, a P4HA2 knockdown significantly decreased cellular invasion and migration in Matrigel. An in vivo subcutaneous xenograft assay in a nude mouse model led to the same conclusions. Since P4HA2 overexpression correlates with higher levels of collagen types I, IV, and VI, a pathway was hypothesized in which P4HA4 promotes an overexpression of the collagen content, which serves as a major ligand for the activation of PI3K/AKT signaling [29]. HSP47 serves as a human chaperone protein for collagen. Recent findings suggest that HSP47 is significantly overexpressed in GBMs and that it promotes GBM stem-like cell survival by modulating the tumor microenvironment through the TGF-β pathway [30].

#### 3.2.2. Fibronectin

Fibronectin (FN) is a high-molecular-weight glycoprotein poorly represented in normal brain tissue ECMs that binds cellular receptors such as integrins and the other components of the ECM, playing an important role in cellular growth, cellular differentiation, migration, and embryonic development. In vitro and in vivo immunohistochemical studies revealed that fibronectin, especially the isoform containing the ED-A and ED-B sequences, and fibronectin cellular receptors are present in almost all glioblastoma microenvironments, especially around the vessels [31,32,33]. In cell cultures, glioblastoma cells showed chemotactic migration towards fibronectin in a dose-dependent manner; moreover, cell adhesion to fibronectin appeared to be dose-related and dependent on glioma invasiveness [34]. In 3D matrix cultures, the depletion of FN by targeted short hairpin RNA expression compromised collective invasion. Similarly, in orthotopic grafts, FN depletion significantly reduced tumor growth and angiogenesis [35,36].

It has been hypothesized that FN-related GBM invasion downstream involves the Integrin B1 fibronectin receptor and the Src kinase/STAT3 signaling pathways [36]. Moreover, recent findings suggest that GBP2, an interferon-inducible large GTPase, is essential in inducing FN expression via the Stat3-pathway [37]. These data suggest the role of fibronectin in regulating in vivo and in vitro glioblastoma cell invasion; moreover, it has been recently proposed as a prognostic biomarker since high FN1 expression appears to be related to poor prognoses [38].

#### 3.2.3. Laminins

Laminins are a large group of glycoproteins consisting of three long polypeptides (the alpha, beta, and gamma chains present in different isoforms) that are not abundant in normal brain tissue and that are mainly present in the basal lamina. The proteins are multifunctional and play roles in development, differentiation, and cell migration, as they can interact with many cell surface proteins. Laminins are abundant in the glioma microenvironment and are mostly associated with the basal lamina of blood vessels, especially in the brain/tumor confrontation zone. In vitro studies revealed that, in the presence of laminins, glioma cell lines form F-actins, form strong and dense stress fibers, and increase the number of pseudopodia on the cell surface, stimulating cell adhesion and invasion. Moreover, during the progression of glial tumors, laminin-9 (alpha4beta2gamma1) is switched to laminin-8 (alpha4beta1gamma1), which is, therefore, considered to be primarily involved in glioblastoma invasion [39]. According to these findings, the overexpression of Laminin isoform-411 (Laminin 8) has been identified to be correlated with higher recurrence rates and the shorter survival of GBM patients. As expected, the depletion of laminin-411 with CRISPR/Cas9 in human GBM cells led to the reduced growth of the resultant intracranial tumors in mice and significantly increased the survival of the host animals by suppressing the Notch pathways compared to mice with untreated cells [40]. Similar in vitro studies have shown that antisense oligonucleotides against both the alpha4 and beta1 chains of laminin-8 are able to significantly block the invasion of cocultures in Matrigel [41]. The debate is on the role of Laminin alpha-5. Recent zebrafish xenograft models with knocked-down Laminin alpha-5 indicate that lama5 discourages glioblastoma cell dispersal and decreases invasion despite previous evidence indicating laminin alpha-5 to be promigratory in in vitro settings [42].

#### 3.2.4. Periostin

The implication of PRO in GBM growth has been enquired in the last decade. Indeed, it was found that the expression of PRO is related to glioma grading and is inversely related to OS. Moreover, in cases of the overexpression of PRO, the genes related to cell migration and proliferation, such as MMP-9, were significantly enriched [43]. Recent evidence suggests p73 to be a main inductor of glioblastoma cell invasion through the direct activation of PRO [44]. It is thought that PRO could stimulate the transforming growth factor β (TGF-β)-induced epithelial–mesenchymal transition via the Akt and Fak signaling pathways [45].

### 3.3. Glycoproteins

#### Tenascin

Tenascins are large hetero- or homohexameric glycoproteins in which the subunits are held together with disulfide bonds. Four members are known in this family: Tenascin-C, -R, -X, and -W. Tenascins are involved in the modulation of cell adhesion, migration, and growth. Tenascin–C is found to be overexpressed in the glioblastoma microenvironment. In vitro, TNC knockdown glioblastoma cell lines were characterized by increased adhesion to the ECM components mediated through the upregulation of the FAK-pathway [46]. Moreover, in the presence of endogenous or exogenous TNC, glioblastoma cell lines expressed an increase in cell migration in a dose-related manner, while no differences in cellular growth were detected. Similarly, mouse xenograft models showed that tumors derived from TNC knockdown cells were less invasive, with tumor cells confined to better-defined tumor borders compared to the control tumors, while tumor size was approximately equal [47]. These data suggest the role of tenascin-C in modulating glioma cell invasion and migration through the ECM without interfering with cell proliferation. 

Recently, it has been proposed that TNC expression in glioma tissue may be promoted by the IL-33-ST2-NFkB pathway [48]. Although the details of the role of downstream Tenascin-C in GBM invasion are not clear up to date, proteases, Cathepsin-B, MMP-12, and the ADAM-9-MAPK8 pathway were found to play a pivotal role in TNC-mediated GBM invasion [49,50,51].

### 3.4. Degradative Enzymes

MMPs are a group of zinc-dependent endopeptidases that degrade several components of the ECM via integrin mediation, participating in tissue structural changes, cell proliferation, and cell migration. At least 23 members of the human MMP family have been identified. GBM cells are known to secrete various MMPs through which they degrade various ECM proteins, including fibronectin, laminins, collagen, and gelatin, promoting cell migration and releasing activated proteins through cleavage [85]. Specifically, a specific subgroup of MMPs (including MMP-1, -2, -7, -9, -11, -12, -14, -15, and -25) was shown to be strictly related to glioma grading and glioblastoma development. In particular, high levels of MMP-9 and MMP-2 were found to be associated with a higher tumor grade, a lesser response to chemotherapy, and a worse survival outcome [52,53,54].

Different pathways of MMP activation have been investigated. The role of the uPA-uPAR pathway in the activation of MMP-9 and MMP-2 in GBMs is well established [55]. uPA is a protease, which is overexpressed in high-grade gliomas, that converts plasminogen to plasmin with a better efficacy when anchored to its receptor, uPAR. Both uPA and plasmin are responsible for MMP activation [56,57]. Moreover, uPA/uPAR, through an interaction with the integrin receptor, has been proven to activate downstream signaling through the activation of FAK, ERK, and Src, which lead to F-Actin assembly, membrane protrusion, and cell migration. Recently, other signaling pathways have been identified. The activation of Sonic Hedgehog signaling is related to an increase in the migration and invasion of GBM cells, which is mediated through the overexpression of MMP-9/-2 via the PI3K/AKT pathway [58]. In a similar way, it has been suggested that even IL-17A might control glioma cell invasiveness by inducing the overexpression of MMP-9/-2 via PI3K/AKT [59]. However, Rictor, a component of the mTOR complex, induces glioma cell migration, increasing MMP-9 expression through the Raf-1-MEK-ERK signaling pathway [60].

Membrane-type MMPs are a subgroup of metalloproteinases that are membrane-associated and have cytoplasmic domains, which may be important in cellular signaling. It has been proven that MT-MMP plays a role in the cleavage of pro-MMP to the active form of MMP-2 [86]. MT1-MMP was found to be involved in the epithelial-to-mesenchymal-transition of glioblastoma cells through pathway signaling, which involves transforming growth factor beta and SNAIL [61]. Similarly, other studies found a correlation between the expression of the MT1-MMP, Beta1-integrin, YAP1 pathways and the grading of gliomas [62].

The subfamily of adamalysins (ADAM proteases) was shown to be overexpressed in glioblastoma cell lines in vitro and in glioblastoma patients and may contribute to cell invasion. ADAM-10 and ADAM-17 are overexpressed in the glioblastoma microenvironment. In vitro studies reported that ADAM10 and ADAM17 inhibition selectively increases glioma sphere-forming cells but not neural stem cell migration and that the migrated GSCs exhibit a differentiated phenotype, suggesting a role in retaining the cells in the tumorigenic environment in an undifferentiated state [63,64].

### 3.5. Physical Forces

Many studies have proven that ECM mechanical changes influence glioma cell invasion, migration, and morphology [65]. Particularly, in vitro studies revealed that, in highly rigid ECMs, tumor cells spread extensively and migrate rapidly, whereas, in lower rigidity ECMs, comparable to normal brain tissue, tumor cells appear rounded and fail to migrate productively [66,67]. Moreover, a variation in matrix stiffness induced the differential expression of enzymes in which HA-synthases and MMP-1 were upregulated in the stiff condition [68]. It has been proposed that tumor-associated mesenchymal stem-like cells could play an important role in glioblastoma ECM remodeling through CCL2/JAK1/MLC2 signaling [69].

Other in vitro studies reported that, in response to hyperosmolarity and hydrostatic pressure, GBM cell lines upregulated the expression of urokinase-type plasminogen activator (uPA) and matrix metalloproteinases (MMPs), promoting cell invasion [70].

### 3.6. Glioma Cell and Microglia Migration and Invasion Patterns

Carcinoma cell invasion is a complex reciprocal process in which cells induce the reorganization of the structure and composition of the ECM, and, in turn, the microenvironment influences cancer cell function, migration pathways, and cell morphology [71].

GBM cells are regulated through several environmental mechanisms that facilitate the spread of these tumors. For example, the invasion pattern of malignant GBMs is associated with the distinct anatomic pathways following the myelinated fiber tracts and blood vessels. In addition to the anatomical and physical aspects, there is accumulating evidence that specific ECM components (such as hyaluronan, vitronectin, and tenascin-C) are unregulated at the border of the spreading GBMs, and this may alter cellular invasiveness. Molecular guidance during cell invasion is often dependent on the ECM, and the underlying mechanism of glioblastoma invasion and the GBM-specific ECM microenvironment represent interesting and potentially meaningful fields of research [65].

In vitro studies, using patient-tissue-derived decellularized ECMs and glioblastoma cell lines, revealed that cancer cells that move through the ECM can be distinguished by their invasion mode. The mesenchymal mode is based on the MMP proteolytic degradation of the matrix, and, in this mode, cells have an elongated morphology and show a polarized extension of the leading edge; additionally, in the ameboid mode, rounded cells tend to migrate in the absence of proteolytic ECM degradation and squeeze through the ECM space [71]. Different ECM compositions have been proven to play a pivotal role in regulating the cell morphology and the migration pathway. For example, in 3D collagen matrices, glioma cells typically show mesenchymal-like migration, whereas, in the presence of HA cells, they assume an ameboid-like pattern [72]. Moreover, GBM cells treated with MMP2/9 inhibitors have a rounded-ameboid mode of invasion, whereas the inhibition of HA synthases (HASs) promotes the morphological transition from a rounded-amoeboid to an elongated-mesenchymal morphology [73]. These findings, together, suggest that the ECM-cell interaction could lead to a switch between these two patterns of migration, enhancing glioma cells’ ability for invasion and representing a mechanism of target therapy escaping.

According to the literature, glioma cell invasion develops preferentially along preexisting tracks such as myelinated axons and blood vessels. Indeed, in vivo studies have shown that the glioma cells that spread along blood vessels and those directly invading the brain parenchyma exhibit different morphological features, the former being spindle-shaped with a single pseudopodium towards the direction of movement and the latter exhibiting multiple pseudopodia with random invasion directions. Moreover, the former exhibits an overexpression of Rho family GTPase activity in contrast with the latter, which exhibits an overexpression of Rac1 and Cdc42 activity [74]. In support of these findings, recent in vitro studies showed that the glioma neurospheres located close to the rods tend to assume a collective strand and to perform fast migration along this physical support, maintaining cell–cell contact, whereas the cells facing the matrix directly exhibit single-cell and random migration in 3D matrices with pseudo vessels recreated using sterile microrods coated with Matrigel [75].

According to recent findings, the nontumoral cells in the glioma microenvironment may reciprocally interact with the ECM components and with glioma cells themselves, representing a further cell migration and invasiveness modulation system. Macrophages/microglia account for up to 30% of the cells in the glioma microenvironment. It is known that macrophages can assume two different forms in tissue repair and, most of all, in ECM remodeling: classical M1-activation, in which the macrophages present an ameboid or round shape and which has been supposed to sustain a proinflammatory role, and alternative M2-activation, in which unipolar macrophages are present and which has a role in antagonizing proinflammatory mediators [76]. In vitro studies showed that microglia can degrade and migrate through the ECM by using a wide range of degradative enzymes. Interestingly, M2-activated macrophages present a higher rate of migration compared to M1-activated macrophages, which is sustained, most of all, by the overexpression of MMP2, Cat-k, and Cat-s [77]. Glioma cells may directly influence the activity of surrounding nontumoral cells with extracellular vesicles, leading, in this instance, to a differentiation of macrophages toward the M2-activated form [78], which, indeed, is the most represented in the glioma microenvironment among the macrophage phenotypes, suggesting a role of activated macrophages/microglia in tumor growth.

Previous in vitro studies have shown that, in presence of microglia, glioma cells show a higher rate of migration and invasiveness. Moreover, such a phenomenon seems to be microglial-specific since replacing microglial with nonmicroglial cells, such as oligodendrocytes or endothelial cells, did not show any significant impact on glioma cell migration. It has been proposed that macrophages/microglia directly affect glioma cell migration through the ECM by secreting MMPs and that they indirectly affect it by promoting the activation of pro-MMP secreted in the microenvironment by glioma cells by means of membrane-type metalloproteases (MT-MMP), which has been proven to be overexpressed in tumor-associated microglia. Among all the different forms of degradative enzymes, MMP-2 seems to play a pivotal role. In fact, in organotypical brain slice models, MMP-2 activity was found to be much higher when glioma cells were cultured in the presence of microglia. Altogether, these data suggest that microglia could play an important role in tumor cell invasion by cooperating with glioma cells themselves in the remodeling of the extracellular matrix, providing a favorable substrate for migration [79]. In support of the data, in vitro studies revealed that glioma cells tend to migrate, in a heterogeneous shape, toward activated microglia-conditioned media and that such migration is sustained through an overexpression of MMP-2 [80,81].

Moreover, not only may the microglia modify the structure of the ECM, but the latter can also have an effect on the former: it has been proven that different components of the ECM could influence microglial activity. Indeed, tumor specimen studies revealed a close relation between the number of macrophages and the Tenascin-C content in the glioma extracellular matrix, suggesting that Tenascin-C may represent a permissive substrate for macrophagic migration in gliomas [82]. Moreover, microglia expressed different morphologies in the Tenascin knockdown glioma xenograft when compared to the control group, exhibiting the first ameboid-like morphology, resembling activated microglia, and the latter resembling inactivated microglia with long and thin processes, pointing out once again the reciprocal interaction between microglial cells and the extracellular matrix. Similarly, mouse xenograft studies revealed that Versican acts as a major ligand for the Toll-like receptors expressed on the macrophage surface, inducing the activation of the latter through an overexpression of MT-MMP [46,83].

In a recent study, the migratory behavior of the microglia and tumor glioma cells at the tumor infiltrative edge was studied and compared to better understand the dynamics of tumor infiltration and, eventually, the reciprocal interactions between the microglia and glioma cells. As reported, in a mouse brain slice model, microglial cells exhibited a migration pattern termed “simple diffusive” characterized by a random walk in a nonrestricted environment, whereas glioma cells exhibited a migration pattern termed “super diffusive” characterized by a persistent directionality of cell migration. At the infiltrative edge, microglial cells present a higher migration speed and a lower directionality compared to the microglial cells located in the peritumoral area, whereas glioma cells present, on average, a higher speed and directionality compared to microglial cells. Moreover, both the microglial and glioma cells exhibit little motility when located further away from the tumor infiltrative edge. Considering these findings, it has been proposed that glioma cells stimulate the activity and motility of microglial cells towards the infiltrative edge and that, in turn, activated microglial cells condition the infiltrative edge microenvironment by modifying the extracellular matrix to reduce the impedance to migration, allowing efficacious glioma cell invasion [84].

## 4. Discussion

The ECM forms a critical and dynamic scaffold that supports the normal brain architecture. It is a complex nonhomogenous structure which physiologically displays a vast and complex interaction with neural and supporting cells. Normal brain tissue has unique components that are not expressed in other tissues. In fact, the brain ECM presents small amounts of fibrous proteins, such as collagen, fibronectin, and laminins, and high amounts of glycosaminoglycans (either bound to proteins, chondroitin sulfate, dermatan sulfate, heparan sulfate, and keratan sulfate, or unbounded in the form of hyaluronan); proteoglycans, called lecticans (versican, aggrecan, neurocan, brevican, and decorin); and glycoproteins, such as tenascin-C.

A large number of studies have proven that the ECM does not only have a mechanical supporting role in the regulation of neural stem cell behavior, neuronal migration, the formation of axonal processes and their myelin sheaths, synapse formation, etc. [4]. Indeed, cells interact with the components of the ECM through membrane receptors (integrins, CD44, BEHAB/brevican, and N-CAM), producing molecular responses and establishing mutual interactions in which cells can modify the microenvironment and vice versa.

**Figure 2:**
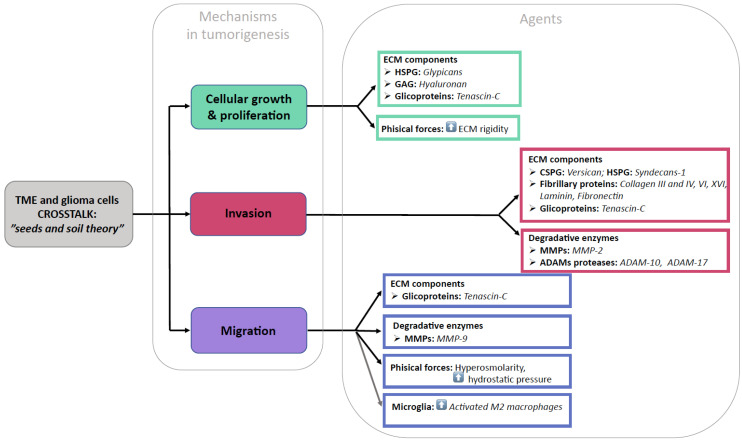
Pictorial summary of our findings.

Functional studies in vitro and genetic studies in mice have provided evidence that the ECM affects virtually all the aspects of nervous system development and function and that it ultimately plays a significant role in all the phases of GBM development and progression. The ECM in GBMs shows significant remodeling compared to the ECM in a healthy brain. Recent evidence highlights that the three-dimensional ECM architecture and its mechanical properties affect the cell behavior in the TME both at the tumoral core and in its periphery. The ECM elements have been shown to display either an attractant or repellant action respective to the glioma cells, microglia, monocytes, macrophages, and stem cells that constitute the TME. We will herein examine the different ECM structural component modifications in GBMs (Figure 2).

## 5. Conclusions

Our systematic review demonstrates that the ECM should not be regarded anymore as a passive scaffold statically contributing to mechanical support in normal and pathological brain tissue but as an active player in tumor-related activity. Further research is necessary to fully understand the clinical implications of these preliminary findings.

## Abbreviations

BEHAB	Brain-enriched hyaluronan-binding protein
CD44	Cluster of differentiation 44
CD280	Endo180
CHI3L1	Chitinase-3-like protein 1
CNS	Central nervous system
COL1A2	Collagen alpha-2(I) chain
CSPG	Chondroitin sulfate proteoglycan
ECM	Extracellular matrix
ED-A	Extra domain A
ED-B	Extra domain B
EGFR	Epidermal growth factor receptor
FAK	Focal adhesion kinase
GAG	Glycosaminoglycan
GBM	Glioblastoma
HSPG	Heparan sulfate proteoglycan
IL-1	Interleukin-1
LAR	Leukocyte common antigen-related
MEK/ERK	Mitogen-activated protein kinase/extracellular signal-regulated kinase
MMP	Matrix metalloproteinase
NFkB	Nuclear factor kappa-light-chain enhancer of activated B cells
N-CAM	Neural cell adhesion molecule
PAI-1	Plasminogen activator inhibitor-1
P4HA2	Prolyl-4-hydroxylase subunit 2
PRISMA-P	Preferred Reporting Items for Systematic Review and Meta-Analysis Protocols
PRO	Periostin
RHAMM	Receptor for hyaluronan-mediated motility
siRNA	Small interfering RNA
TGF-β	Transforming growth factor beta
TME	Tumor microenvironment
uPA	Urokinase-type plasminogen activator
uPAR	Urokinase-type plasminogen activator receptor
